# Hypnotism as She Is Taught in Chicago

**Published:** 1896-11

**Authors:** 


Hypnotism as She is Taught in Chicago.—In Chicago there is a School of Psychology, under the management of Dr. Herbert A. Parkyn. They teach and practice hypnotism. There is a clinic held at certain hours, and the cases "treated" are published in the Hypnotic Magazine mentioned elsewhere.

Hypnotism as taught and practiced by the school seems to be a universal panacea for the ills of man; they stop at nothing. Ætiology and pathology cut no figure; everything is treated by the one "remedy" "*suggestion.*" It matters not whether it be a neurosis—(and one would naturally suppose those diseases not characterized by change of structure of any organ would be susceptible, more or less, to mental influence), or whether it be a disease in which structural change has taken place—even cancer,—these people claim to cure it by "suggestion;" which seems to be a kind of a process by which the patient is convinced that he is a fool; that he is mistaken, that there is nothing the matter with him; that he has only to believe that he is well to be made well. He must discredit the most positive evidences to the contrary; racking pain, for instance, or visible destruction of tissue, as in cancer sometimes,—or change in structure, as in granular lids,—(cases cited in the magazine before us.) If the patient will "have faith." Aye, there's the rub. How can one have faith in something which discredits one's every sense? And, if one can "have faith" at the suggestion of another, thereby giving that other "power," why go around the elbow to get to the thumb? Why not request him—the patient to have "power" at once, and tell his senses that they are all mistaken; and thus banish all evidences of disease? It strikes us that there is precious little difference in the teachings and practices of this "school" and the "Christian Scientists."

And—by the bye—the head of the school, Dr. Parkyn, he who reports these "clinics" for the Hypnotic Magazine, says that hypnotism is in no sense, sleep,—which if true—makes it misnomer. He says:

"The popular opinion regarding the curative effects of hypnotic suggestion seems to be that unless the patient loses consciousness or passes into what spiritists call "the trance state," no benefit will result. *It takes a whole week* sometimes to get this foolish notion out of a patient's brain, and to implant in its stead the simple fact that a state of drowsiness is all that is necessary to *effect a cure* [italics ours.—Ed.] of any nervous disease or functional disorder by suggestion. If the patient is moderately intelligent, he will see the force of the argument that he must assist the operator by cultivating a state of passivity, and encouraging the drowsy feeling, but too often the patient returns to her friends after the treatment with the observation, "Well, I didn't sleep. He thought I did, but I didn't. He couldn't send me to sleep!"

"Takes a whole week" does it, to "implant the simple fact, ("*fact*" mind you) that a *state of drowsiness* is *all that is necessary effect* a *cure* of any nervous disease or functional disorder by suggestion. It would take a couple of whole weeks to convince some persons; well, for instance, this fellow—reported along with a lot of others—embracing skin disease, chronic constipation, dysmenorrhœa (whether obstructive or functional makes no difference); blepharitis, hemorrhoids, convulsions [?] doesn't say of what kind or origin; well, the whole catalogue;—

"R. S., colored. aged 29, was treated for stammering and for cigarette habit. First treatment, patient passed into a state of profound sleep (nothing very remarkable in a negro going to sleep in an easy chair; I'll bet he was 'possuming); then, too, it was not necessary," (and by the bye, perhaps this accounts for the failure, both to "cure" and to "convince," etc.) "Suggestion that he should not smoke any cigarettes that day. Next morning he reported having tried only two cigarettes, but threw them away unsmoked; "didn't taste right," (Boss) he said. Given suggestion that if he tried to smoke a cigarette that day it would nauseate him. Reported next morning that he was unable to smoke a cigarette during the preceding afternoon. Patient then remarked that he would come back in an hour (see you later—nit); got upon his bicycle, and has not since returned." "I should not pronounce his case cured," says the doctor; in all seriousness, and without a glimmer of a sense of the ludicrous. If the doctor's overcoat didn't disappear along with his patient, he is to be congratulated. Suggested him out of notion,—p'raps.

Hypnotism will some day be understood and intelligently used; used with benefit in more ways than one, may be. It is a "force," known to man so far only by its manifestations, as was once electricity. To "*pooh-pooh*" it and say "humbug," is only to show one's ignorance and intolerance; but we have not gotten far enough along with the study of this force to chain it to a chair or a bed-post in a hospital and make it do service in all cases without discrimination. It is something of an advance however, on the "faith cure," or "Christian Science." One set employ it empirically, and claim results; the other invoke it with some conception of its origin power and scope; and it bears about the same relation at present to the practice of medicine as electricity did thirty years ago. At that time electricity was only employed in medicine by quacks, and to become identified with the use of electricity was to become ostracised in the profession. Electro-therapeutics is now a little better understood, and is legitimate. The day will come when hypnotism will be a powerful arm in the equipment of a physician, if in no other way, as an anesthetic. We believe when this force is better understood, and can be more intelligently employed, it will be useful; but as to its ever being made to "cure" tumors, cancers, consumption, wounds, granular lids, as positively claimed now by the Chicago enthusiasts, by "suggestion,"—well—why not go a little further and "suggest" to an old fellow, whose teeth are gone, whose hair has all dropped out, whose eye is dim, and whose footstep falters on the brink of the grave—that he is

*mistaken*; that he is not old at all;—that he is a mere youth;—in fact, that it is all his imagination about his not having any teeth to eat the corn cake, and n'ere an eye for to see. In fact, rejuvinate him; make him live forever. It strikes us that this is as possible as to "suggest" away cancer or granulated lids.

Then, indeed, will Ponce de Leon's spring have been discovered—at Chicago; the *elixir vitae*—sought for by the alchemists of antiquity—found,—in Chicago; Brown-Sequard put to blush with his—"juices"; and a plausibility be given to Bulwer's "Strange Story."—Great is Chicago.

